# Enhancement of Astaxanthin Bioaccessibility by Encapsulation in Liposomes: An In Vitro Study

**DOI:** 10.3390/molecules29081687

**Published:** 2024-04-09

**Authors:** Li Pan, Haojie Meng, Jiaqi Li, Zongjie Liu, Dongsheng Zhang, Zhengyang Liu, Qian Zhao, Fei Xu

**Affiliations:** Oil and Food Engineering Technology Research Center of the State Grain and Reserves Administration/Key Laboratory of Henan Province, Henan University of Technology, Zhengzhou 450001, China; panli215@haut.edu.cn (L.P.); hjmeng22@163.com (H.M.); 17503825393@163.com (J.L.); liuzj9420@163.com (Z.L.); donsen2004@163.com (D.Z.); a1223371130@163.com (Z.L.); 15238099328@163.com (Q.Z.)

**Keywords:** astaxanthin, liposomes, encapsulation, release, lipolysis, bioaccessibility

## Abstract

Astaxanthin was encapsulated in liposomes by a thin layer dispersion and ultrasound method using soybean phospholipid. The digestion properties of liposomes for encapsulating astaxanthin were investigated in light of particle size, size distribution, zeta potential, and microstructure during in vitro digestion as a function of time. These results exhibited that the average particle size increased gradually with liposomal vesicles retained round shapes and a fairly uniform distribution after passage through the simulated gastric fluid digestion. The result revealed that astaxanthin-loaded liposomes were stable in low pH conditions. It was also found that the mixed micelles formed in a simulated intestinal fluid. The zeta potential of astaxanthin-loaded liposomes had a decrease in negativity after digestion. In comparison with free astaxanthin, there was an appreciable increase in the bioaccessibility of astaxanthin after encapsulation in liposomes. This enhancement can be attributed to more soluble astaxanthin in the mixed micelles for astaxanthin-loaded liposomes. It indicated that the barrier of the liposomal bilayer could inhibit astaxanthin fading and leaking after encapsulation in liposomes. These results provide useful information for designing more stable delivery systems in the gastrointestinal tract and improving the bioaccessibility of lipophilic nutraceuticals.

## 1. Introduction

Astaxanthin (AST), chemically named 3,3′-dihydroxy-β, β′-carotene-4,4′-dione, is one of the keto-carotenoid pigments, which come from aquatic animals, including salmon, shrimp, and algae [1]. In recent years, AST has attracted remarkable attention because of its prevention of lipid peroxidation, impartation of color, and powerful antioxidant activity [2]. AST exhibits superior antioxidant activity compared to other carotenoids, such as lutein, lycopene, β-carotene, α-tocopherol, and zeaxanthin [3]. Further, AST has displayed other health benefits, including protection of neuronal and cardiovascular, anti-inflammatory effects, anti-diabetes, and antitumor activity [4]. Given these potential biological functions, there is considerable interest in the utilization of AST as a nutrient in pharmaceuticals, nutraceuticals, and supplements. Nonetheless, because of the highly conjugated and unsaturated structure of AST, it is prone to oxidize, degrade, and isomerize under high temperature, oxygen, light, pH, and organic solvent conditions [5]. During food production, AST is susceptible to degradation, especially when removed from its biological matrix and exposed to the chemical conditions of the industry [6]. The degradation of AST not only leads to the loss of its biological functions and sensory properties but also to reduced nutritional values and color. Furthermore, AST is soluble in most organic solvents, whereas it is insoluble in water. Thus, AST usually has a relatively low bioaccessibility due to its low water solubility and poor stability, resulting in limited development and expansion of its practical applications. For this reason, various strategies have been developed to improve the stability and solubility of AST. AST has been incorporated into diverse carrier systems involving emulsions, liposomes, nanoencapsulation, nanodispersions, complexes of hydroxypropyl with β-cyclodextrin, edible oils, and nanostructured lipid carriers [7].

Among the carriers, liposomes are introduced as a drug-delivery vehicle first and applied widely in the nutrition and food industries later [8]. Liposomes are bilayer vesicles formed by self-assembling amphiphilic lipids in an aqueous environment, whose structure is similar to a biological membrane [9]. In recent years, liposomes have received more and more attention owing to their non-toxicity, amphipathic characteristics, biocompatibility, biodegradability [10], sustained release properties, as well as the ability to enclose both lipophilic and hydrophilic substances [11,12]. Based on the information provided, it can be concluded that liposomes have outstanding advantages over other delivery systems. In our previous work, storage and thermal stability, antioxidant activity, water dispersibility, and sustained release profile of encapsulated AST in liposomes were remarkably enhanced [13,14]. However, to our knowledge, there have been few studies investigating the digestion behavior of AST encapsulated in liposomes. Digestion occurs in the gastrointestinal tract and involves a series of complicated reactions and processes that influence the uptake of astaxanthin. Obviously, it is highly instructive to study potential gastrointestinal digestion processes of AST-loaded liposomes, especially for AST bioaccessibility.

Thus, the objective of this study was to extend our previous work and to provide a more comprehensive understanding of the digestion behavior of liposomes for encapsulating AST and enhancing the bioaccessibility of AST. It has been reported that physiological conditions and sequential occurrence of events during digestion in the human gastrointestinal tract could be simulated using an in vitro digestion model [15]. In vitro study denotes a tool for assessing the physicochemical processes of digestion and the bioaccessibility of encapsulated substance. In this study, we investigated the gastrointestinal digestion processes of AST-loaded liposomes in vitro. The particle size, particle size distribution, zeta potential, and microstructure of AST-loaded liposomes were measured during in vitro oral-gastrointestinal digestion. The bioaccessibility and color of encapsulated AST by liposome during digestion were evaluated with free AST used as a control.

## 2. Results and Discussion

### 2.1. Changes in Particle Size and Size Distribution during In Vitro Digestion

**Figure 1:**
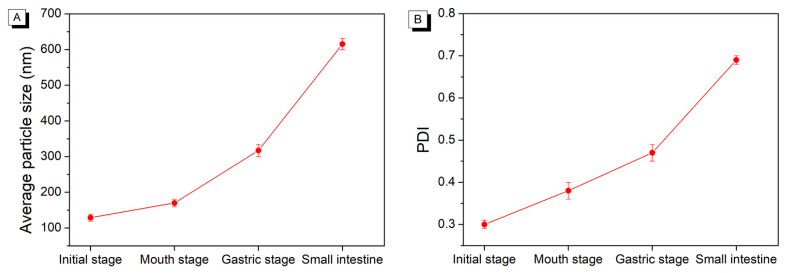
Change in average particle size (**A**) and polydispersity index (PDI, (**B**)) of astaxanthin-liposomes (AST-liposomes) during in vitro digestion.

**Figure 2:**
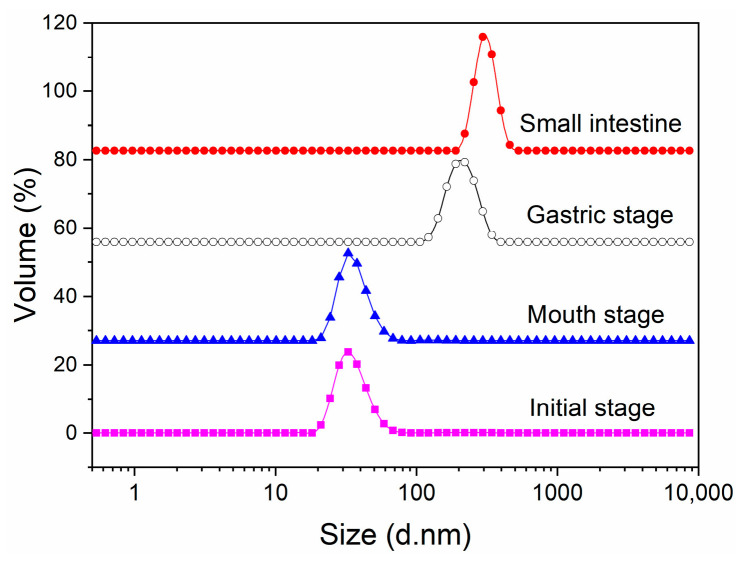
Change in size distribution of AST-liposomes during in vitro digestion.

As depicted in Figure 1A, the average particle size of initial AST-loaded liposomes was 128.90 ± 9.63 nm. The polydispersity index (PDI) is a measurement of the size distribution of liposomes, ranging from 0 to 1.0 [16]. A high PDI value indicates a broad size distribution, which corresponds to a less homogeneous particle size [17]. The initial AST-loaded liposomes presented a PDI of 0.30 ± 0.01, as shown in Figure 1B, indicating a fairly uniform distribution. Compared to the initial AST-loaded liposomes, it exhibited slight changes in particle size or size distribution after being incubated in simulated saliva fluid. Additionally, the particle size and size distribution of AST-loaded liposomes increased while passing through from the mouth to the gastric digestion stage. At the same time, it was notable that the size distribution of AST-loaded liposomes still retained unimodal after the simulated gastric fluid digestion stage (Figure 2). The outcomes suggested AST-loaded liposomes were stable under in vitro gastric digestion conditions owing to the protection of liposomal membrane from fusion and aggregation at low pH values. It was also found that the average particle size of AST-loaded liposomes increased gradually from the initial stage to the simulated intestinal fluid stage. The possible cause could be the fusion, aggregation, and swelling of liposomal particles. On the other hand, free fatty acids released from the phospholipid bilayer might aggregate and adsorb on the surface of liposomes [18]. After incubation in simulated intestinal fluid, it should be noted that the average particle size of AST-loaded liposomes increased steeply from 317.00 ± 17.33 nm to 615.40 ± 16.12 nm. Pancreatin is a compound that consists of pancreatic lipase, trypsin, and amylase. Phospholipids in liposomes were hydrolyzed by the catalytic action of pancreatic lipase, which released lysophospholipids and fatty acids during the small intestine digestion stage. Meanwhile, the AST released from liposomes, bile salts, phospholipids, and fatty acids formed larger mixed micelles due to the strong surface-active nature of bile salts, resulting in a considerable increase in particle size.

### 2.2. Changes in Zeta Potential during In Vitro Digestion

**Figure 3:**
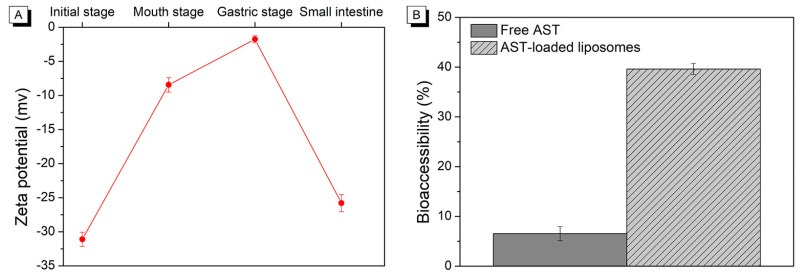
Change in zeta potential of AST-liposomes (**A**) and AST bioaccessibility of free AST and AST-liposomes during in vitro digestion (**B**).

Zeta potential can be used as an indicator for assessing the physical stability of liposomes [19]. A higher absolute value of zeta potential corresponds to a stronger surface charge for liposomes. This increased charge enhances the repelling force between particles, preventing them from aggregating and flocculating [20]. The zeta potential of AST-loaded liposomes was monitored during various stages of the simulated GIT. As presented in Figure 3A, the zeta potential of initial AST-loaded liposomes was −31.10 ± 1.04. The high zeta potential for initial AST-loaded liposomes could be ascribed to the interactions of soybean phospholipid and AST. The dipole tropism is generated due to the interaction of the choline of the polar head group in soybean phospholipid with the hydroxyl group in AST, leading to an increase in surface charges of liposomes [21]. The zeta potential of AST-loaded liposomes appeared less negative at −8.44 ± 1.07 mV after incubation in simulated saliva fluid, which might be attributed to the association of positive species onto the liposome surface. After the gastric digestion stage, the zeta potential became considerably less negative (−1.76 ± 0.52 mV). It might be due to the plenty of hydrogen ions in the simulated gastric fluid, which could effectively neutralize anions and decrease the negative charge. Following exposure to the digestion stage in the small intestine, the zeta potential of AST-loaded liposomes displayed a highly negative charge of −25.80 ± 1.26 mV. It could be related to the phospholipids hydrolyzation of liposomes, thereby generating the lysophospholipids and free fatty acids. The existence of anionic components involving lysophospholipids, bile salts, and free fatty acids in mixed micelles resulted in a highly negative charge on liposomes.

### 2.3. Changes in Microstructure during In Vitro Digestion

**Figure 4:**
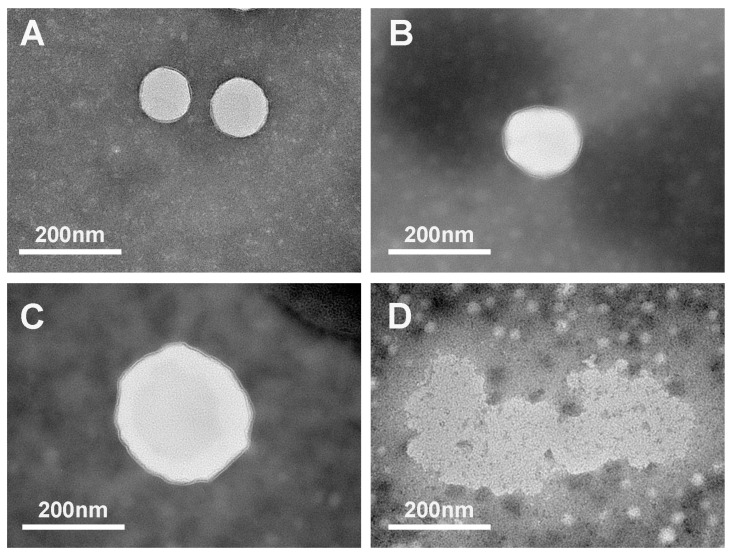
Change in microstructure (determined by transmission electron microscopy) of AST-liposomes during in vitro digestion: initial stage (**A**), mouth stage (**B**), gastric stage (**C**), and intestine stage (**D**).

TEM was used to observe the changes in the microstructure of AST-loaded liposomes during passage through the simulated GIT. As exhibited in Figure 4, the initial AST-loaded liposomes without digestion clearly showed intact structure and spherical shape without any roughness, whose particle sizes were approximately in the range of 130 to 150 nm. The outcome was consistent with that of the dynamic light scattering particle size measurement mentioned above. The microstructure of AST-loaded liposomes was minimally influenced and appeared mostly spherical in shape with slight roughness and irregularity as they passed through from the initial stage to the gastric digestion stage. It revealed that AST-loaded liposomes were stable at low pH values during simulated gastric fluid digestion, which could be due to the hydrogen bonding between AST and the lipid bilayer, thereby decreasing the membrane fluidity and maintaining the integral structure. It also presented that the particle size of AST-loaded liposomes during the three stages of digestion was larger than that of the initial stage. The results obtained using dynamic light scattering further corroborate the conclusion. Following incubation in simulated intestinal fluid, the presence of big particles with a coarse and dense surface morphology was observed, indicating that these particles were likely mixed micelles. This phenomenon could be attributed to the hydrolysis of phospholipids in liposomes catalyzed by pancreatic lipase, resulting in the aggregations of liposomes. Furthermore, mixed micelles were formed as a result of the presence of bile salts. In addition, many small liposomal particles were observed after incubation in simulated intestinal fluid due to the strong emulsification of bile salts, which could embed and split the liposome vesicles into small particles before the generation of mixed micelles.

### 2.4. The Bioaccessibility of Astaxanthin

Bioaccessibility is defined as the proportion of a compound that is released from its food matrix in the gastrointestinal tract and becomes available for intestinal absorption. Carotenoids form mixed micelles in the small intestine by combining with free fatty acids or monoglycerides, phospholipids, bile salts, etc. Subsequently, the mixed micelles are transferred to the epithelial cells of the small intestine in a variety of ways, resulting in their absorption by the body [22].

The available proportion of AST released into the mixed micelles for absorption following in vitro digestion was defined as the bioaccessibility of AST [23]. In this section, simulated GIT was utilized to compare the bioaccessibilities of free AST (physically mixed with blank liposomes) and AST encapsulated in liposomes. The blank liposomes represent the liposomes without AST. As illustrated in Figure 3B, the bioaccessibility of AST encapsulated in liposomes (39.61 ± 1.13%) was appreciably higher than that of free AST (6.54 ± 1.42%). According to the report [24], the solubility of hydrophobic nutrients in the mixed micelles generated by bile salts and lipid digestion products was a key challenge in improving its bioaccessibility. As mentioned above, our previous study has demonstrated that encapsulation remarkably improved the water solubility of AST. In our previous work, most of the free AST was present as an insoluble precipitate and suspended in water [14]. As a result, AST encapsulated in liposomes was more bioaccessible than free AST due to its easier transfer to the mixed micelles and higher solubility in mixed micelles. Additionally, the incorporation of AST into the liposomal membrane with enhanced stability prevented AST from interacting with the surroundings, thereby reducing the degradation and leakage of encapsulated AST [25]. However, when free AST was not protected by liposomes, it was subjected to stomach digestion, resulting in increased leakage and chemical degradation compared to encapsulated AST, which was protected from digestive enzymes until small intestine digestion. It suggested that less AST was soluble in the mixed micelles for free AST, resulting in lower bioaccessibility.

### 2.5. Changes in Color Characteristics during In Vitro Digestion

The chemical stability of the emulsion during storage can be assessed by measuring the color change. The emulsion will fade during storage, such as the decrease in color intensity and the increase in brightness value [26]. Preparing AST solution and AST-loaded liposomes at the same concentration and monitoring color changes throughout gastrointestinal digestion could assess the stability of AST during the digestive process.

The changes in the color of AST during passage through digestion were evaluated using monitoring the color indexes (L*, a*, b*, and Δ*E*) and summarized in Table 1. The value L* is a measurement of lightness (higher value represents a lighter color); value a* is a measurement of redness (A higher positive value implies a redder color, while a higher negative value indicates a greener color.) [27]; value b* is a measurement of yellowness (A greater positive value indicates a more yellow color, whereas a higher negative value represents a bluer tint) [28].

Δ*E* is mainly influenced by the color lightness (L*), which could express how far apart two colors are in the color space [29]. As shown in Table 1, there was a similar variation trend of color parameters for encapsulated AST and free AST during digestion. A progressive increase in value L*, as well as a decrease in values a* and Δ*E* was observed, which was indicative of color fading for the two samples throughout the digestion stage. As compared to free AST, the lower color value L*, higher values a* and Δ*E* implied the darker color for the encapsulated AST from mouth to small intestine stage digestion. In other words, there was less color fading for encapsulated AST than free AST during digestion. Meanwhile, it was also discovered that the color fading velocity of free AST was substantially faster than that of encapsulated AST during digestion. Particularly after the gastric stage of digestion, there were significant differences in the color parameters L*, a*, and Δ*E* between AST encapsulated and free AST. These results clearly implied that encapsulated AST was more stable than free AST under the simulated gastrointestinal conditions, which could be due to the protection of liposomes. Once encapsulated by liposomes, the liposomal bilayer could effectively prevent AST fading and leaking as a barrier.

## 3. Materials and Methods

### 3.1. Materials

Shenyang Tianfeng Biopharming Co., Ltd. (Shenyang, China) provided the soybean phospholipid (soybean phosphatidylcholine = 98%). Shanghai Yanyu Trading Co., Ltd. (Shanghai, China) supplied the AST (98%). Analytical grade reagents and chemicals were used. Ultrapure water was utilized to prepare all solutions and liposomes.

### 3.2. Preparation of Astaxanthin-Loaded Liposomes

AST-loaded liposomes were fabricated by the film dispersion with the supersonic wave method on the basis of our current work [30]. A volume of 5 mL of chloroform was subjected to dissolve 1 mg of AST and a lipid consisting of soybean phospholipid and cholesterol (5:1, *w*/*w*). After removing the chloroform from the solution, it was hydrated with 25 mL of 0.05 mol/L phosphate-buffered solution (pH 7.4) including 100 mg of Tween 80, while being vortexed for 30 min at 50 °C. The produced suspension was then homogenized using probe ultrasonic equipment (JY96-IIN, Ningbo Xinzhi Biotechnology Co., Ltd., Ningbo, China) for 4 min at 100 W in an ice-water bath. The AST-loaded liposomes were poured into brown bottles filled with nitrogen and stored away from light at 4 °C.

### 3.3. Simulated Gastrointestinal Digestion

The simulated gastrointestinal tract (GIT) model was adopted to assess the digestion behavior of AST-loaded liposomes. The three-stage gastrointestinal tract (GIT) model consisted of the mouth, stomach, and small intestine stages [31].

Mouth stage: The simulated saliva fluid (pH 6.8) was fabricated by adding 0.6 g of mucin, 0.202 g of KCl, and 1.594 g of NaCl to 1 L of ultrapure water. About 10 mL of initial liposomes was blended with simulated saliva fluid (40 mL). The mixture was then adjusted to pH 6.8 by 1.0 M NaOH and shaken at 100 rpm for 10 min in the water bath (37 °C).

Gastric stage: 2 g of NaCl, 3.2 g of pepsin, and 7 mL of HCl were added to 1 L of ultrapure water, and the pH was then adjusted to 1.2 by 1.0 M HCl to fabricate a simulated gastric juice with pH 1.2. The samples extracted from the mouth stage were then blended with the simulated gastric fluid at a volume ratio of 1:1. The pH of the mixture was then adjusted to 1.2 by 1.0 M NaOH and shaken at 100 rpm for 1 h in the water bath (37 °C).

Small intestine stage: The simulated intestinal fluid (pH 7.4) was fabricated by adding 5.16 g of bile salt, 4.76 g of pancreatin, 6.8 g of KH_2_PO_4_, and 250 mL of 0.1 M NaOH to 1 L of ultrapure water and adjusting the pH to 7.4 by 1 M NaOH. The pH of samples extracted from the gastric stage was adjusted to 7.4 before adding the simulated intestinal fluid. Then, the mixture was poured into the simulated intestinal fluid at a volume ratio of 1:1 and shaken in the water bath (37 °C) for 2 h at 100 rpm.

For liposomes both before and after exposure to each stage of simulated GIT, the particle size, particle size distribution, zeta potential, and microstructure were examined.

### 3.4. Particle Size, Size Distribution, and Zeta Potential Measurements

The particle size, size distribution, and zeta potential of liposomes after each digestion stage were estimated using dynamic light scattering (Nano-ZS90, Malvern Instruments Ltd., Worcestershire, UK). The angle of scattering was fixed at 90° at 25 °C [32]. Prior to evaluation, the liposomes were diluted 200 times with ultrapure water to prevent multiple scattering effects. Each sample was carried out in triplicate.

### 3.5. Microstructure Study

The microstructure of the liposomes after each digestion stage was carried out using transmission electron microscopy (TEM). Before evaluation, one drop of the sample was diluted two times with a phosphate-buffered solution (pH 7.4). The diluted samples were deposited onto a copper mesh, including a supporting film (Beijing Zhongjingkeyi Technology Company, Ltd., Beijing, China), and afterward stained using a phosphotungstate solution (2%) [33]. The superfluous samples were wiped off with filter paper after 10 min. The air-drying copper grid was observed under indoor temperature using a TEM (HT7700, Hitachi, Tokyo, Japan).

### 3.6. Bioaccessibility Measurement

The bioaccessibility of AST was evaluated when the samples passed through the fully simulated GIT model based on the study [34]. 10 mL of raw digestion content was centrifuged at 16,000 rpm at 4 °C for 40 min after digestion in vitro process. The samples were separated into a supernatant phase at the top and a subside phase at the bottom after centrifuge. The resulting supernatant was collected as the micellar fraction, where AST is dissolved. 5 mL of the micellar fraction was filtered using a syringe filter (0.45 μm PP) to displace any residuary larger particles. The filtrate or 5 mL of raw digestion content was mixed with 5 mL chloroform through vortexing in a water bath at 30 °C and centrifuged at 3000 rpm for 5 min, respectively. The bottom chloroform phase (including dissolved AST) was gathered, and the upper phase was added with 5 mL chloroform. The above steps were repeated twice. Then, the two bottom chloroform phases gathered are combined together. The concentration of AST in the supernatant was analyzed using an ultraviolet spectral photometer (TU-1810, Beijing Puhua General Instrument Co., Ltd., Beijing, China) at a wavelength of 492 nm and quantified according to a standard curve, in which chloroform was applied for blank. The bioaccessibility of AST was obtained as follows:

(1) $$Bioaccessibility\left(\%\right)=\frac{\;C_{micelle}}{C_{raw\;digest}\;}\times 100$$

where *C*_micelle_ and *C*_raw digest_ are the concentrations of AST in the micellar fraction and in the overall raw digest content after exposure to the simulated GIT, respectively.

### 3.7. Color Measurement

The tristimulus color coordinates (L*, a*, and b*) of the liposomes after each digestion stage were assessed using a hand-held colorimeter (CR-400, Konica Minolta, Chiyoda City, Japan) according to the study [26]. The liposomal sample was poured into a transparent flat-bottomed cuvette; afterward, the measurement equipment of the colorimeter was pressed against the cuvette surface, and the color was monitored. Each test was performed in triplicate. The total color difference (∆*E*) of the liposomes when passed through each digestion stage was calculated using the following equation [35]:

(2) $$∆E=\sqrt{∆L^{{*}^{2}}+∆a^{{*}^{2}}+∆b^{{*}^{2}}}\;$$

### 3.8. Statistical Analysis

All experimental data reported in this study were performed at least thrice and exhibited as mean value ± standard deviation (SD). SPSS software (version 23.0, IBM Corp., Chicago, IL, USA) was utilized to estimate the statistical analysis (*p* < 0.05) through one-way analysis of variance (ANOVA). Based on Duncan’s multiple comparison measure, the significant differences among the treatments were determined.

## 4. Conclusions

This study displayed the gastrointestinal digestion properties of AST-loaded liposomes. The particle size, size distribution, zeta potential, and microstructure of AST-loaded liposomes changed throughout digestion. These results suggested that AST-loaded liposomes were stable under in vitro gastric digestion conditions. This could be attributable to hydrogen bonding between AST and the lipid bilayer, thereby decreasing the membrane fluidity and maintaining the integral structure. After incubation in simulated intestinal fluid, mixed micelles were generated because of the strong surface-active nature of bile salts. The bioaccessibility of AST was enhanced markedly by encapsulation in liposomes, which might be due to the reason that AST encapsulated in liposomes is more bioaccessible than free AST. The color measurement indicated that encapsulated AST was more stable than free AST during digestion because of the protection of liposomes. The findings might offer a vital insight into the digestion properties of AST encapsulated in liposomes during in vitro digestion.

## Figures and Tables

**Table 1:** The color formation of free astaxanthin and AST-liposomes during in vitro digestion.

Sample	Digestion Stage	L*	a*	b*	ΔE*
Free AST	Initial stage	13.13 ^a^ ± 0.03	3.13 ^d^ ± 0.03	5.15 ^d^ ± 0.04	82.31 ^d^ ± 0.03
	Mouth stage	13.83 ^c^ ± 0.02	2.68 ^c^ ± 0.04	4.90 ^c^ ± 0.01	80.00 ^c^ ± 0.04
	Gastric stage	13.70 ^b^ ± 0.03	1.88 ^b^ ± 0.04	4.41 ^b^ ± 0.04	75.36 ^b^ ± 0.04
	Small intestine	15.88 ^d^ ± 0.03	1.50 ^a^ ± 0.03	3.97 ^a^ ± 0.04	74.00 ^a^ ± 0.04
AST-liposomes	Initial stage	13.22 ^a^ ± 0.01	3.06 ^d^ ± 0.02	5.27 ^d^ ± 0.04	82.04 ^d^ ± 0.01
	Mouth stage	13.65 ^c^ ± 0.02	2.85 ^c^ ± 0.03	4.57 ^c^ ± 0.03	81.59 ^b^ ± 0.02
	Gastric stage	13.53 ^b^ ± 0.01	2.41 ^b^ ± 0.03	4.03 ^b^ ± 0.04	81.07 ^c^ ± 0.01
	Small intestine	15.48 ^d^ ± 0.01	2.11 ^a^ ± 0.02	3.43 ^a^ ± 0.06	79.73 ^a^ ± 0.01

Values are expressed as the mean ± SD. Different letters in the same column indicate significant differences (*p* < 0.05).

## Data Availability

The original contributions presented in the study are included in the article, further inquiries can be directed to the corresponding author.
